# Evidence for hypoxia increasing the tempo of evolution in glioblastoma

**DOI:** 10.1038/s41416-020-1021-5

**Published:** 2020-08-27

**Authors:** David Robert Grimes, Marnix Jansen, Robert J. Macauley, Jacob G. Scott, David Basanta

**Affiliations:** 1 School of Physical Sciences, Dublin City University, Dublin 9, Ireland; 2 Cancer Research UK/MRC Oxford Institute for Radiation Oncology, Gray Laboratory, University of Oxford, Old Road Campus Research Building, Off Roosevelt Drive, Oxford, OX3 7DQ UK; 3 Departments of Endoscopy and Pathology, University College London Hospital, London, UK; 4 Department of Pathology, H. Lee Moffitt Cancer Center and Research Institute, Tampa, FL USA; 5 Departments of Translational Hematology and Oncology Research and Radiation Oncology, Cleveland Clinic, Cleveland, OH USA; 6 Integrated Mathematical Oncology, H. Lee Moffitt Cancer Center and Research Institute, Tampa, FL USA

**Keywords:** Cancer microenvironment, Cell division, Computational science, Cancer microenvironment, Cell division

## Abstract

**Background:**

Tumour hypoxia is associated with metastatic disease, and while there have been many mechanisms proposed for why tumour hypoxia is associated with metastatic disease, it remains unclear whether one precise mechanism is the key reason or several in concert. Somatic evolution drives cancer progression and treatment resistance, fuelled not only by genetic and epigenetic mutation but also by selection from interactions between tumour cells, normal cells and physical micro-environment. Ecological habitats influence evolutionary dynamics, but the impact on tempo of evolution is less clear.

**Methods:**

We explored this complex dialogue with a combined clinical–theoretical approach by simulating a proliferative hierarchy under heterogeneous oxygen availability with an agent-based model. Predictions were compared against histology samples taken from glioblastoma patients, stained to elucidate areas of necrosis and *TP53* expression heterogeneity.

**Results:**

Results indicate that cell division in hypoxic environments is effectively upregulated, with low-oxygen niches providing avenues for tumour cells to spread. Analysis of human data indicates that cell division is not decreased under hypoxia, consistent with our results.

**Conclusions:**

Our results suggest that hypoxia could be a crucible that effectively warps evolutionary velocity, making key mutations more likely. Thus, key tumour ecological niches such as hypoxic regions may alter the evolutionary tempo, driving mutations fuelling tumour heterogeneity.

## Introduction

While genetic alterations are the fuel of somatic evolution, the tumour micro-environment is the key contributor to the selection process that could be described as its engine.^1^ The tumour micro-environment consists of multiple elements that impact tumour cell fitness, thus shaping selection for key cancer phenotypes^1–3^ that characterise tumour progression. Oxygen is a key element of the micro-environment, long known to play a pivotal role in patient prognosis, with ample evidence confirming that tumour oxygenation has important implications for patient outcome and treatment response.^4,5^ Clinically, poorly oxygenated tumours respond significantly worse to treatment than well-oxygenated regions,^4,6^ but in addition, oxygen is a known selection pressure, favouring aggressive cancer cell phenotypes characterised by certain traits, including the capacity to endure harsh environments and to migrate beyond the tissue from whence they arose.^7^ Such clones gain the ability to proliferate and survive in hypoxic environments,^8^ suggesting that hypoxia can initiate metastasis. While stable and well supplied in healthy tissue, tumours tend to have highly heterogeneous microscopic oxygen supply, a direct consequence of the erratic vasculature encouraged by tumour angiogenesis.^9,10^ Improving understanding of the interplay between the oxygen micro-environment and cancer evolution is of paramount importance to advancing therapy,^11–14^ yet it is notoriously difficult to probe this question with experimental tools alone. Mathematical modelling allows us to explore the consequences of various assumptions and informs the understanding of what is clinically observed,^15,16^ and better understands the spatio-temporal dynamics to which the study of fixed tissue or molecular biology is typically blind. In this work, we take a combined mathematical model and histology approach to ascertain whether hypoxic regions select for clonogenic cells and whether an increase in the stress of the tumour cells therein increases evolutionary tempo relative to normoxic regions.

## Methods

It has become increasingly recognised that the integration of mathematics and clinical as well as experimental data in oncology can yield novel insights that are clinically relevant.^15^

A hybrid discrete-continuous cellular automata (HCA) approach^17^ of a proliferative hierarchy was developed, simulating evolutionary dynamics of clonogenic cells in heterogeneous oxygen environments. Image analysis and next-generation sequencing was performed on human glioblastoma sections, triple stained with haematoxylin and eosin (H&E), Ki-67, and *TP53* mutation markers with regions of necrosis delineated by pathological examination. All clinical aspects of this study approved by the Moffitt Cancer Center IRB, with informed consent given for the anonymised database samples analysed in this work.

### Clonogenic cell model outline

**Fig. 1:**
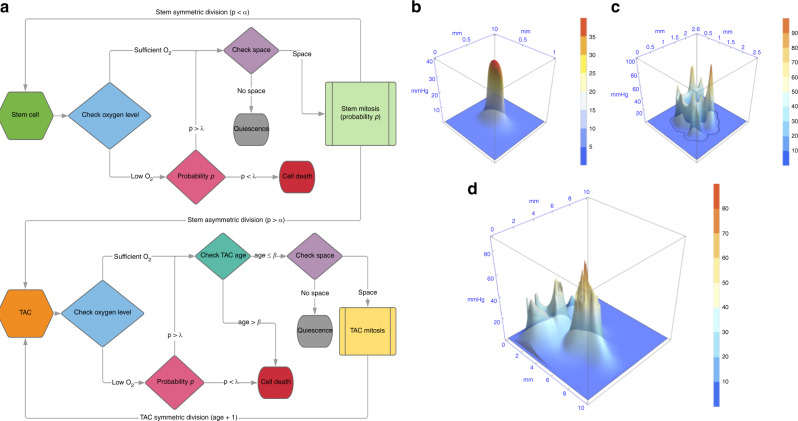
Model schematic and example oxygen maps. **a** Cell fate decisions per each cellular update are determined by the flow charts displayed. Note that the non-italicised p in this schematic refers to probability rather than oxygen tension. Example static heterogeneous oxygen maps with **b** 1, **c** 15 and **d** 357 vessels.

To explore stem cell dynamics in a heterogeneous oxygen environment, we used an agent-based HCA model built upon the framework developed previously^18^ with modification. The schematic is outlined in Fig. 1a. Briefly, it consists of clonogenic cells that can symmetrically divide (with probability *α*) into two identical stem cells, or asymmetrically into a clonogenic daughter and a daughter transient amplifying cell (TAC) with probability 1 − *α*, provided there is free space for the cells to occupy. Clonogenic cells are effectively immortal unless killed by anoxia; TACs divide to other TACs only, and these cells can only undergo *β* divisions before undergoing apoptosis. TAC daughter cells inherit the divisional age of their parent TAC. An alternative explanation of our assumptions is that any cancer cell can give rise to another cancer cell. Modelling suggests that the assumptions made have serious implications for tumour growth,^19^ and it is worthwhile to consider both options. To implement the assumption that all cells would proliferate, the simulation was also run with *α* = 1 so no TAC cells would emerge. To factor in the influence of the oxygen micro-environment, simulations were run with a variety of oxygen maps, with the addition of conditions for hypoxia-mediated death. These maps were simulated from previously derived vascular maps/oxygen kernels,^9^ scaled up to illustrate typical oxygen heterogeneity. Figure 1b–d depicts simulated oxygen maps derived from 1, 15, and 357 vessel configurations, respectively. In regions below a critical oxygen threshold *p*_C_, cells have a probability *P*_D_ of death per time-step, simulated with both the Heaviside switch function and oxygen-dependent death function, as outlined in Supplementary Appendix S1. The HCA model was run considering these oxygen maps, following the evolution of cancer cells in the micro-environment, recording not only cell position but also the divisional age of cells (i.e. the number of total divisions in their life history). Divisional age was taken as a proxy for mutational risk, as cells that undergo more divisions have increased chance of producing an offspring with a clinically relevant mutation (e.g. conferring increased therapeutic resistance or metastatic potential). Each grid position is assumed to be the width of one cell. For simplicity, no cellular compression was assumed. The model was run 1000 times over each oxygen maps outlined and output analysed. Simulation parameters are given in Supplementary Material S1.

### Analysis of clinical data

**Fig. 2:**
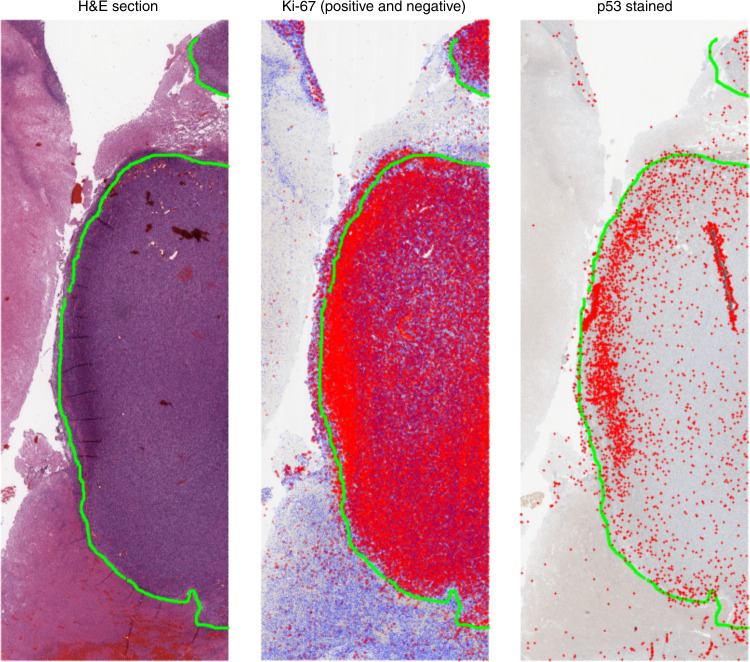
Co-registration and cell detection analysis. A necrotic boundary is marked on the H&E slide by the pathologist (marked here by the green line). On the Ki-67 stain, cells that meet the threshold for Ki-67 positive are marked by red dots and those below the threshold by blue dots. Finally, p53-positive cells are marked by red (+) symbols on the final stain. The region shown above encompasses an area of 87.52 mm^2^ (15.67 mm × 5.58 mm).

Human glioblastoma sections were obtained from patient biopsy samples. For each tumour, three adjacent sections were prepared as follows: (1) H&E; (2) immunohistochemistry (IHC) for the proliferation marker Ki-67 and (3) IHC for p53 protein. While overexpression of the latter can sometimes be interpreted as a surrogate for TP53 gene mutation and gene dysregulation in a number of cancers,^20–24^ it is chiefly an indicator of physiological cellular stress. Gene sequencing was also performed on the sections to determine whether TP53 gene mutation was present or not, with all clinical aspects of this study approved by the Moffitt Cancer Center IRB. To quantify cellular features, microscopy was performed at high resolution using the Digital Pathology Leica Biosystems Aperio system. Images were taken at ×20 magnification, yielding digital images of the sections with 1 pixel corresponding to 0.504 μm. Regions of necrosis were identified by histological examination on the H&E slide and marked by a specialised neuropathologist (R.M.) using the Aperio *Imagescope* software. These annotations were extracted as XML files with the coordinates of necrotic boundaries. While explicit oxygen concentration cannot be determined from this experimental data, a major benefit of using glioblastoma sections is that necrosis in these cancers is strongly associated with hypoxia, so that necrotic boundaries could be treated as a reliable proxy for hypoxia even without explicit oxygen concentrations. This assumption is justified in more detail in the ‘Discussion’ section. A co-registration algorithm was written for this work, which identifies features in adjacent slides and aligns the images. Once images were co-registered, cells staining both positive and negative for Ki-67 were identified automatically on the Ki-67 slide and p53-positive cells on the p53 slide. The image analysis code determines the distance from the coordinates of each cell centre to the nearest boundary of identified necrosis, recording the minimum distance to necrosis for each cell of interest. An example of the co-registration and cell identification technique is shown in Fig. 2. A full description of the image registration algorithm, image analysis protocol and sample code is included in the Supplementary Material S1.

#### Evolutionary pressure of hypoxia

From the quantification of clinical data discussed above, we can now investigate the hypothesis that cells in the hypoxic niche are at higher risk of mutation. For a clonogenic cell, we assume that the rate at which mutations are accumulated per unit time, *γ* is related to the division rate *g* and the intrinsic risk of mutation per division, *r*_d_. It follows that for multiple divisions, $\gamma =1-{(1-{r}_{\mathrm{d}})}^{g}$. When *r*_d_ is small, the binomial approximation allows us to *γ* ≈ *r*_d_*g*, and thus the probability of a clonogenic cell acquiring a mutation with time *t* is given by Poisson statistics as $M(t)=1-\exp (-{r}_{\mathrm{d}}gt)$. Under conditions of high cellular stress, as those in the hypoxic niche, we can expect a higher intrinsic probability of mutation per division *r*_s_, where *r*_s_ > *r*_d_, reflecting the evolutionary pressures of the micro-environment on cellular evolution.^25^ We define the mitotic rate in the hypoxic niche as *g*_s_ and thus

1 $${M}_{\mathrm{s}}(t)=1-\exp (-{r}_{\mathrm{s}}{g}_{\mathrm{s}}t).$$

If hypoxia leads to an increase in mutation rates, then we would expect *P*_s_(*t*) > *P*(*t*), ∀*t* > 0, where *P*_s_(*t*) is the probability of a mutation under hypoxia and *P*(*t*) the probability of mutation under normoxia. Determining this requires us to probe the mitotic status of the hypoxic niche. There is evidence that cells in the hypoxic niche respond to stress by entering a state of quiescence,^26–28^ markedly reducing their rate of mitosis (*g*_s_ ≪ *g*). In this case, it is possible that *M*_s_(*t*) < *M*(*t*), which would imply that hypoxia is not a selection pressure for evolutionary change. Alternatively, if there is evidence that cells in the hypoxic niche continue to undergo the same approximate rate of mitosis as cells in well-oxygenated regions, then it follows that mutations will be much more likely to arise in hypoxic niches. There is good biological evidence that hypoxia diminishes DNA repair and elevates mutagenesis.^29^ Using the histological analysis outlined, the distribution of both p53-positive (physiologically stressed) cells and mitotically active Ki-67-positive cells were quantified in different regions to determine whether the mutational risk was elevated under hypoxia, and results contrasted with model predictions.

## Results

### Model-derived results

#### Oxygen-dependent distribution of clonogenic cells

**Fig. 3:**
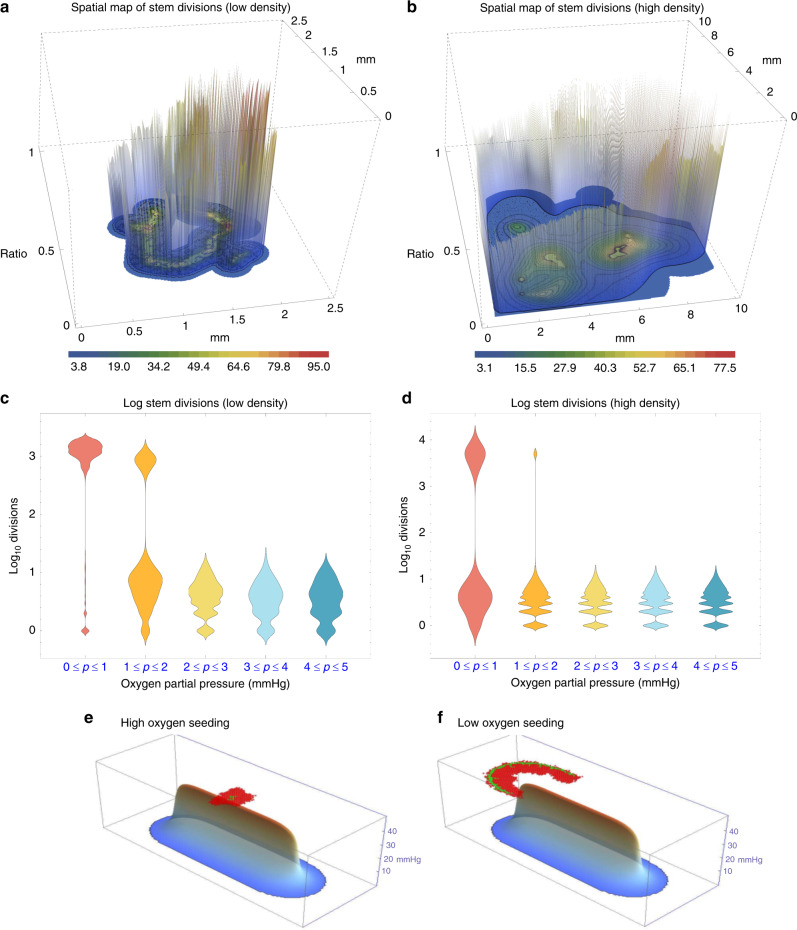
Simulation results of hypoxia effects. Clonogenic division in **a** low-density oxygen map and **b** high-density oxygen map. Colour bars indicate oxygen partial pressure, and vertical bars indicate divisions of clonogenic cells, normalised to the maximum number of divisions in the simulation. **c**, **d** show respective clonogenic cell divisions higher under hypoxia. In **e**, **f**, clonogenic cells are shown in green and TACs in red, with *β* = 15 after 10,000 time-steps. **e** depicts cells initially seeded in a high oxygen environment and **f** initial cell seeded on the hypoxic niche. In **e** the firewall effect of long-lived TACs can be clearly seen, whereas in **f** clonogenic cells proliferate along the anoxic ridge, yielding an ‘edge creep’ effect, allowing invasion invade along the hypoxic ridge.

Figure 3a–d depicts the stratification of clonogenic cells relative to oxygen concentration. High division of clonogenic cells was directly associated with low oxygen conditions for all configurations, with clonogenic cells on anoxic borders undergoing far more divisions than well-oxygenated cells. Qualitative observation of the HCA reveals that this increase in divisional age is secondary to cyclic instances of birth and death as cells place daughters into areas of extreme (lethal) hypoxia. As daughters die, clonogenic cells continue to divide as they sense free space. The same trend was observed if all cells were presumed to be clonogenic, with visions on the anoxic border markedly upregulated.

#### Hypoxic niche as a metastatic avenue

Figure 3e, f depicts the impact of seeding an initial clonogenic cell in oxygen-rich versus hypoxic environments. Previous authors^30^ have shown that clonogenic cells seeded in high oxygen can experience a ‘firewall’ effect, where long-lived TACs impede invasion potential. This simulation suggests that this firewall is overcome when clonogenic cells colonise hypoxic borders, allowing cells to ‘creep’ along the edges of the hypoxic niche. This leads to marked differences in clonogenic population; for the simulation in Fig. 3, high-oxygen seeding with *β* = 15 led to only 13 ± 4 stem cells after 10,000 steps. By contrast, low-oxygen seeding yielded 254 ± 25 clonogenic cells in the same interim.

### Clinical data analysis

**Fig. 4:**
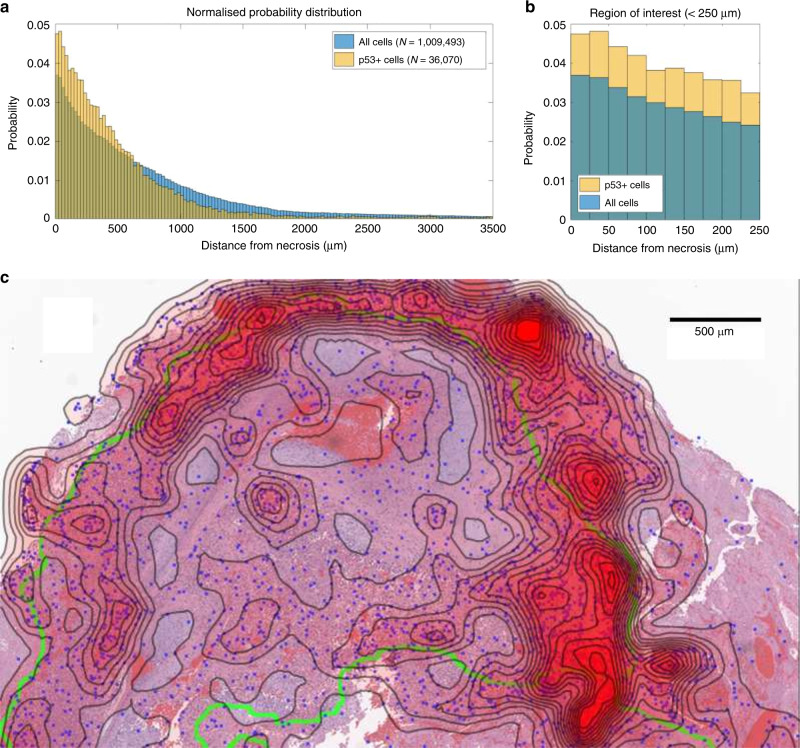
Physiological evidence of hypoxia effects in glioblastoma samples. Pooled data from 23 regions of 9 patient glioblastoma samples after image analysis depicting (**a**) distribution of p53-stained cells versus Ki-67-stained cells relative to known necrotic borders. **b** Probability distributions for stained cells close to necrosis. **c** An example from a patient glioblastoma histologic section. Physiological p53 stress detected by image analysis is illustrated by blue dots overlaid on the histology section. Green lines depict pathologist-marked necrosis; contour lines with red opacity show the probability density of p53-positive cells (calculated from a Sheather–Jones smoothing-kernel distribution function). Near necrotic regions, the probability of finding stress markers increases relative to non-necrotic zones. The mitotic rate appears constant throughout the tissue, suggesting that these regions are more likely to give rise to mutations. Physiological stress indicates potential topography of evolutionary velocity. A decoupled version of the figure is available in Supplementary Material S1.

Clear necrotic borders were ascertained in 23 sections from 9 patients from the Moffitt Cancer Center, in sections ranging from 0.72 to 108.14 mm^2^. Image analysis was performed to determine cells that were both positive and negative for Ki-67, and for cells positive for p53 mutations, and determine their minimum distance from the pathologist-specified necrotic boundary, as outlined in Supplementary Methods. Probability density of spatial distribution from known necrosis for all these data is shown in Fig. 4, in bins corresponding to the width of two cells (25 μm). There was no statistical difference in the distribution of cells both positive and negative for Ki-67 relative to necrosis (two-sample Kolmogorov–Smirnov (KS) test *p* = 0.5668, KS test statistic 0.0802), and accordingly, these are grouped together. Conversely, p53 mutation-positive cells are far more likely to be found near necrotic regions, with a markedly different distribution than grouped Ki-67 cells (two-sample KS test *p* = 1.21 × 10^−7^, KS test statistic 0.2941). Gene sequencing showed no indication of *TP53* mutation, strongly implying that the p53-positive-stained cells resulted from hypoxia-driven physiological stress.

#### Analysis of evolutionary risk

To determine whether cells in the hypoxic niche displayed greater quiescence, the fraction of Ki-67-positive cells over the sum of Ki-67-positive and -negative cells in each 25 μm bin from 0 to 2 mm bin was calculated. This fraction was calculated at 0.2124 ± 0.0104, indicating that the proportion of mitotic cells in each bin was relatively constant and that *g*_s_ ≈ *g*. By contrast, p53 staining was markedly increased close to regions of hypoxia (see Supplementary Material S1 for correlation data), suggesting strongly that cells under physiological stress continued to undergo unrestricted mitosis. This suggests that cells in the peri-necrotic niche have increased mutational risk relative to well-oxygenated cells.

#### Hypoxia and evolutionary tempo

The clinical data show an increase in physiological stress associated with hypoxia. Figure 4 depicts a histological section stained with H&E. In the figure, cells staining strongly positive for p53 mutation as detected by the image analysis in the p53 section are superimposed at their corresponding positions, marked by blue dots. Regions of clear necrosis as demarcated by the neuropathologist (R.M.) are outlined in green on this image. From the spatial map of p53 mutant cells, a probability distribution function for these points in space was ascertained by employing a Sheather–Jones data smoothing kernel. Contour lines of p53 stress density were superimposed over the image, with greater line density denoting increased abundance of p53 staining cell, with a red opacity effect also superimposed to show the highest density of p53 staining cells corresponding to contour lines of concentration. As can be seen, the highest density of stressed cells tends to lie on or close to the line of representing the necrotic anoxic boundary, illustrating stress near the necrotic hypoxic boundaries. This echoes the phenomena predicted in our simulations, with the resultant map yielding likely topography of evolutionary velocity.

## Discussion

Evidence to date suggests that hypoxia selects for aggressive and metastatic phenotypes. In this work, we have investigated the hypothesis that hypoxia influences speed and evolutionary potential of cancer, acting as a potential strong selection pressure for subclonal evolution as defined in recent works by other researchers.^31^ We present mathematical evidence that this impact goes beyond selecting for certain phenotypes more adaptable to low oxygen levels. As we present evidence that proliferation is not impeded under hypoxia, this would suggest that hypoxia may directly modulate the tempo of somatic evolution, so that the speed of somatic evolution is likely significantly increased near the anoxic edges surrounding areas of necrosis. To draw a physics analogy, the presence of hypoxia appears to ‘warp’ evolutionary velocity, effectively creating a region of increased rate of potential mutation acquisition, micro-environmentally mediated evolutionary hotspots.

We used our HCA agent-based computational model to arrive at these conclusions, indicating that clonogenic cell division was substantially more pronounced in regions of hypoxia, as illustrated in Fig. 3.

Our simulations also revealed that the hypoxic niche facilitates migration of clonogenic cells along low oxygen regions. Other authors have found long-lived TAC cells effectively limit cancer growth, acting as a firewall when these offspring cells are sufficiently long-lived.^30^ This model recapitulated that behaviour in well-oxygenated environments, but found it to be broken down around anoxic niches. Clonogenic cells colonised necrotic niches, even when simulated TAC cells were long-lived (*β* > 15). This ‘edge effect’ suggests that the hypoxic niche acts as a conduit to cellular infiltration, effectively changing the way cells interact.^32^ While further biological evidence is required to confirm this, it raises a previously unforeseen potential consequence of hypoxia for tumour evolution.

Histopathological data from glioblastoma patients were examined to challenge in silico predictions and to determine whether modelled behaviour was consistent with it. Image analysis on sectioned regions from glioblastoma patients strongly suggested that cells in hypoxic niches do not undergo any noticeable quiescence, displaying the same fraction of Ki-67 proliferation marker as well-oxygenated regions. This was observed even in areas with clear markers of severe physiological stress, suggesting tumour cells proliferate unimpeded by the stressful conditions they find themselves in, increasing their risk of mutation.

Given the limited nature of histological data, we are mindful not to overly infer, but this investigation provides yielded no evidence of reduced proliferation in severely stressed hypoxic regions in these glioblastoma samples. Combined with the modelling findings, this suggests that mitosis is not only unimpeded, but it is likely increased adjacent to anoxic zones. If this is the case, and more cell divisions occur on this periphery, this would perhaps explain the appearance of pseudopalisading necrosis, a hallmark feature in these brain tumours where regions of profound hypoxia are surrounded by an accumulation of tumour cells.^33^ More than this, the concurrence of these findings suggests that the combination of increased division and hypoxic stress makes these regions crucibles for driving mutations, perhaps explaining why hypoxia is such a risk factor for detrimental mutation. The model finding of increased proliferation at anoxic edges might also be related to what is observed with pseudopalisading necrosis, but further investigation would be needed to confirm this beyond doubt.

This is not unprecedented—insensitivity to signalling and persistent proliferation are of course hallmarks of cancer.^34^ There is ample evidence that hypoxia elevates mutagenesis,^29,35,36^ and modelling results in this work suggest a mechanistic reason why cells in the hypoxic niche would be far more likely to acquire mutations than well-oxygenated cells, leading to the eventual emergence of metastatic- or treatment-resistant types.

It is important to note that there is evidence that hypoxia can, under different circumstances, either reduce or up-regulate proliferation through metabolic adaptation.^37–39^ This is likely to depend on properties of the tumour cell, and specific microenvironmental considerations. In tumour spheroids, for example,^4,40^ mitosis is seen down to very low oxygen tensions. This suggests that the biological reality is complicated by other factors, and results from this work and others cannot be carelessly generalised

It is worth noting that findings of this work are largely independent of whether the stem cell hypothesis is considered or not. This is important, as the generality and exact properties of cancer stem cells are heavily debated, but has been clearly demonstrated in some cancers.^41–46^ As the same result is seen under either assumption, it is agnostic to whether this hypothesis is accepted or not. Increased division by clonogenic cells in the hypoxic niche elevates the probability of a cell acquiring a mutation (and ultimately metastatic potential), in part explaining hypoxia’s strong correlation with emergence of metastatic phenotypes and poor prognosis.^7^ The biological evidence here is, of course, indirect due to the limitations of staining analysis, and specialised experiments would be needed to fully test the hypothesis. Importantly, however, observed data are in accordance with simulation predictions based on mechanistic principles, suggesting a fertile avenue for future exploration.

The model presented in this work is a simple agent-based model. This mode cannot capture all the complexity of the underlying biology in glioblastoma, but approximates likely behaviours emergent in different cancers. In the model, cells can either be killed off in the hypoxic zones or, in the case of TACs, undergo apoptosis after *β* divisions. This prompts the question of whether small amounts of random death might change the trends observed. To test this, simulations were also run with random death.

For biologically reasonable estimates, results were similar to that presented here, illustrated in Supplementary Material S1. There are a number of limitations to our approach. In regards to modelling, chiefly that the model exists on a 2D grid rather than true 3D space, without consideration of cell motility.^47^ Increased detail and consideration of such factors could improve how precise the model is, but we do not expect main conclusions to be challenged in light of these considerations.

One major attraction of using glioblastoma sections is that there is ample evidence that regions of necrosis are hypoxic, and reference to this can be found in Supplementary Material S1. CA-IX immunostaining was also performed on some of the cases in this work, which confirmed that peri-necrotic regions were indeed hypoxic. That diffusion-limited hypoxia gives rise to necrosis has long been observed in human tissue and experimental models,^4^ and there is a known reciprocal relationship between p53 and hypoxic path.^48^ Necrotic borders in this work are almost certainly hypoxic, but for future investigations, quantifying oxygen gradient may yield further insight into the implications for tumour evolution.

A number of caveats have to be kept in mind when interpreting histological data; 2D histology is at best an approximation of complex 3D behaviour, and can be sometimes misleading.^9^ Defining necrosis robustly was also challenging—while straightforward to demarcate clear regions of necrosis, ambiguous sections were excluded from the analysis. Accordingly, the extent of necrosis may in some instances be an underestimate. Even so, a number of suitable sections were unambiguously identified in the patient data, with over a million individual cells. In this volume of data, we expect general patterns to become apparent even with confounding influences of 2D data.

This work presents modelling evidence that the oxygen micro-environment plays a fundamental role in ‘warping’ the evolutionary velocity of cells under its influence. We also present clinical data that adds support to this hypothesis. Combined, this work highlights the importance of the tumour micro-environment not only in selecting for certain phenotypes but also in regards to the velocity and dynamics characterising its somatic evolution. Hypoxia itself is already detrimental for treatment efficacy,^4^ and this work further suggests that this could be compounded by the ability of this environment to select for phenotypes displaying both increased treatment resistance, evolutionary and metastatic potential. This suggests that hypoxic zones are of substantial pathological interest in terms of tumour evolution, and be a fruitful avenue for future investigations.

## Supplementary information

S1 supplementary

## Data Availability

Simulation parameters and details available in the manuscript and Supplementary Material. Further details of the data analysed in this work are available from the corresponding author on request.
