# Supplementary material for: Evidence for hypoxia increasing the tempo of evolution in glioblastoma
Source: Br J Cancer. 2020 Aug 27;123(10):1562–9. doi: 10.1038/s41416-020-1021-5 (PMC7653934; doi:10.1038/s41416-020-1021-5)
Supplement: Supplementary file 1 — S1 supplementary [file 41416_2020_1021_MOESM1_ESM.pdf]

Evidence for hypoxia increasing the tempo of evolution in glioblastoma - Supplementary material S1

David Robert Grimes[1,2*], **Marnix Jansen**[3], **Robert J. Macauley**[4], **Jacob G. Scott**[5*], **and David Basanta**[6*]

[1]School of Physical Sciences, Dublin City University, Dublin 9, Ireland
[2]Cancer Research UK/MRC Oxford Institute for Radiation Oncology, Gray Laboratory, University of Oxford, Old Road Campus Research Building, Off Roosevelt Drive, Oxford OX3 7DQ, UK
[3]Departments of Endoscopy and Pathology. University College London Hospital, London, UK
[4]Department of Pathology, H. Lee Moffitt Cancer Center & Research Institute, Tampa, Florida, USA
[5]Departments of Translational Hematology and Oncology Research and Radiation Oncology, Cleveland Clinic, Cleveland, Ohio, USA
[6]Integrated Mathematical Oncology, H. Lee Moffitt Cancer Center & Research Institute, Tampa, Florida, USA
[1]* contributed equally to the work. Correspondance: davidrobert.grimes@dcu.ie — david@cancerevo.org

ABSTRACT

Supplementary material **S1** for paper.

Materials and Methods

Patient sample data

Table 1 lists details of the patient sample data used in this work, including the number of cells of each type identified in the section. Figure 1 shows clear correlation between distance from necrotic borders and probability of p53 mutation staining, suggesting physiological stress in these regions consistent with sustained hypoxia.

Table 1. Analysis of experimental Glioblastoma sections

Patient	Sub-section	Area	Ki-67 +	Ki-67 -	P53 +	TP53 mutation
1	i	108.14 mm^2	57,498	208,789	4217	-
	ii	87.42 mm^2	53,435	132,068	3991	-
2	i	27.21 mm^2	13,709	63,052	3072	-
	ii	21.10 mm^2	5814	57,689	1516	-
3	i	25.82 mm^2	10,419	55,213	2722	-
	ii	10.08 mm^2	4794	20,108	1983	-
	iii	18.62 mm^2	13,580	33,186	4547	-
	iv	15.83 mm^2	5090	53,798	275	-
	v	4.93 mm^2	2249	14,333	120	-
4	i	4.09 mm^2	2028	9569	360	-
5	i	2.17 mm^2	1956	4349	1676	-
6	i	2.61 mm^2	1085	6125	37	-
	ii	1.27 mm^2	500	3039	33	-
	iii	5.18 mm^2	2736	12,905	179	-
	iv	0.72 mm^2	385	1935	7	-
	v	2.89 mm^2	943	5985	252	-
7	i	16.10 mm^2	14,072	39,848	4373	-
8	i	10.88 mm^2	4275	10,845	1659	-
	ii	26.77 mm^2	14,406	17,757	4600	-
	iii	4.47 mm^2	2565	11,025	164	-
9	i	3.98 mm^2	1719	8090	92	-
	ii	3.79 mm^2	1810	7930	149	-
	iii	4.52 mm^2	1566	14,490	46	-
-						-

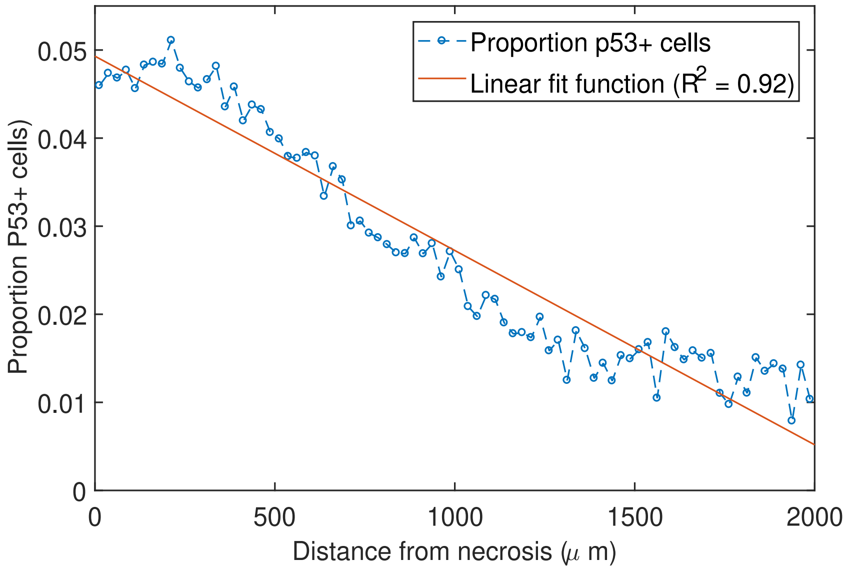

Figure 1. Evidence of Strong physiological stress near necrotic regions By contrast, mitotic fraction did not vary with distance from necrosis.

Modeling considerations

Guide to assumptions and equations

- Our model assumes a heterogeneous tumor made of two subpopulations: clonogenic cells capable of tumor initiation and differentiation (CSCs) and transit amplifying cells (TACs).
- CSCs can replicate indefinitely while TACs have a finite replicative potential of β divisions after which cells die.
- CSCs can divide either symmetrically (with probability α) or asymmetrically (where the two daughter cells will be CSC and TAC, with probability $1-\alpha$). TACs only divide symmetrically resulting in two TACs.
- Space is discretized into a grids up to 1000 x 1000. Time is also discretized into timesteps of one average cell doubling time.
- The micro-environment is determined by heterogeneous oxygen maps derived from our previous work[1].
- Cells (both CSC and TAC) in low oxygen grid points ($p \leq p_C$) have probability $P_D(p)$ of dying on every time-step. We also assume that proliferation is not impacted by O_2 supply.
- Cells (both CSC and TAC) divide any time that space is available and remain quiescent when there is none.

Key equations

Oxygen maps were derived from a previously published oxygen kernel for vascular maps, where partial pressure p at a distance d from a vascular point of radius r_o is given by

$$p = \frac{a\Omega s_L}{3D}\left(\frac{\sqrt{r_n^2-d^2}}{3}(2r_o^2-8r_n^2)+2r_n^3\log\left(\frac{r_n-\sqrt{r_n-d^2}}{d}\right)\right) \tag{1}$$

where D is oxygen diffusion constant in water, r_n is the diffusion distance of oxygen in a specific tissue and Ω and s_L are constants, as previously outlined and omitted here for brevity[1]. The probability (P_D) of a cell dying in a low oxygen niche ($p \leq 0.5$mmHg) was modeled by two methods. The first of which was a Heaviside step function, so that

$$P_D = \begin{cases} 0.5, & p \leq 0.5 \text{ mmHg} \\ 0, & p > 0.5 \text{ mmHg}. \end{cases} \tag{2}$$

The second form allows us to capture the possibility that death probability is dependent on oxygen partial pressure, we employed a Poisson-like death function of

$$P_D(p) = 1-\exp(-k_D p) \tag{3}$$

where k_D is a constant.

Additional parameter table for simulations

Table 2. Simulation parameters

Parameter	Value
Symmetric division probability α	0.25
Asymmetric division probability $(1-\alpha)$	0.75
TAC replications before apoptosis β	6[2]
Critical oxygen threshold p_C	0.5 mmHg[3,4]
Cell diameter	12.5 μm[4,5]
Poisson-like death function constant k_D	1.368 mmHg $^{-1}$ †

†Chosen so that $P_D(0.5 \text{ mmHg}) = 0.5$

Random cell death

In the main paper, model results were presented assuming a 'no-random-death' assumption. In this framework, stem cells were effectively immortal and could only die under hypoxic conditions. TAC cells could die under hypoxia, or after undergoing β divisions. It is worthwhile to check whether the results observed in the modeling work hold if random cell death of both stems and TACs is factored in. Supplementary figure 2 shows the impact of this for random death per cell per time step r_d of 0.02 and 0.05 respectively in the low density rat tumor for 5000 time steps. In the former case ($r_d = 0.02$), results are very similar to those in the main work. In the latter case, the general trend is seen albeit with much reduced probability. This is because this high biologically improbable value for r_d has a tendency to wipe out colonies; the chances S of a cell surviving for n iterations is $S = (1 - r_d)^n$. If $r_d = 0.02$, then a cell can live through $n = 149$ time steps before its survival chances falls to below 5%. By contrast, when $r_d = 0.05$, a cell has less than 5% chance of survival by 59 time steps. Even with these exaggerated dynamics, highly replicating stem cells were still much more likely on the necrotic niche, suggesting modeling results are robust.

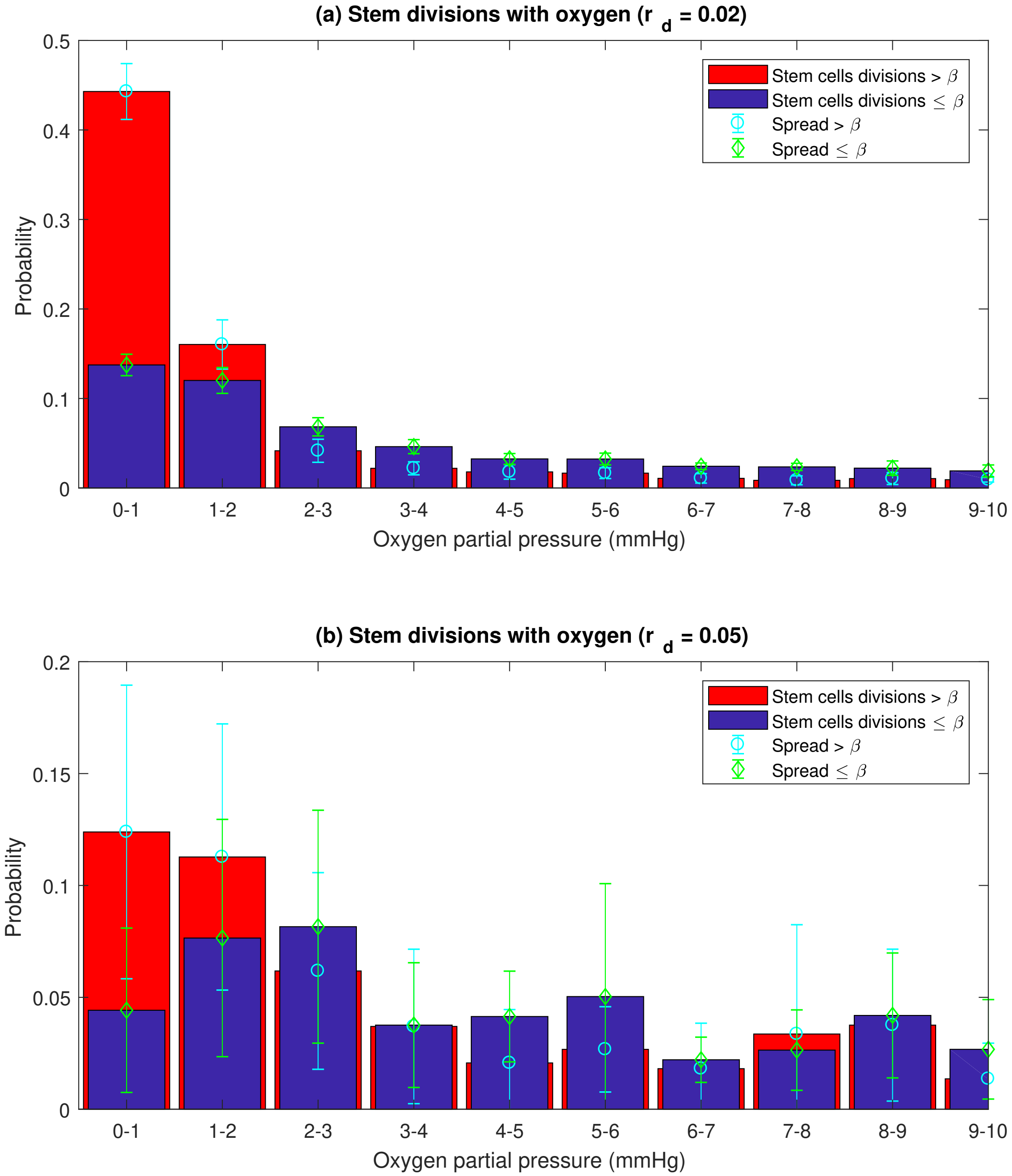

Figure 2. Stem cell distribution with oxygen with random death in model.

Evidence that necrosis in glioblastoma is hypoxic

Evidence to date strongly suggests necrotic regions in glioblastoma are hypoxia mediated[6–11].Pseudopalisading necrotic cells in particular are known to be hypoxic, displaying dramatic up-regulation of hypoxia inducible factor-1[11].

Image thresholding

For p53 analysis, it was important to sufficiently threshold the image so that only unequivocal cells were observed. The following algorithm was used to determine a punishing threshold, and apply it to p53 images.

- Read in red channel of p53 image.
- Convert image to grid of doubles (converts pixel values to values between 0 and 1).
- Invert image so P53 spots have high intensity.
- Find the mean pixel intensity in image, m_{val}.
- To reduce false positives, set threshold to a multiple n of m_{val}, where $n > 1$.
- Binarize the image to this threshold.
- Clear any border pixels.
- Remove any small objects of less than 70 pixels.
- Find centroids of remaining objects.
- Draw over original image.
- Visually inspect, adjust n as required.

For our p53 images, $n = 3$ was sufficiently high to gate ambiguous cells. This punishing cut-off might have meant that some p53 staining cells were under counted, but even using lower thresholds (such as n = 2.5) yielded the same trend, provided $n >> 1$. An illustration is provided in figure 3. As this threshold was quite punitive, it should only select for the most unequivocally staining cells. This suggests weaker physiological up-regulation isn't skewing analysis.

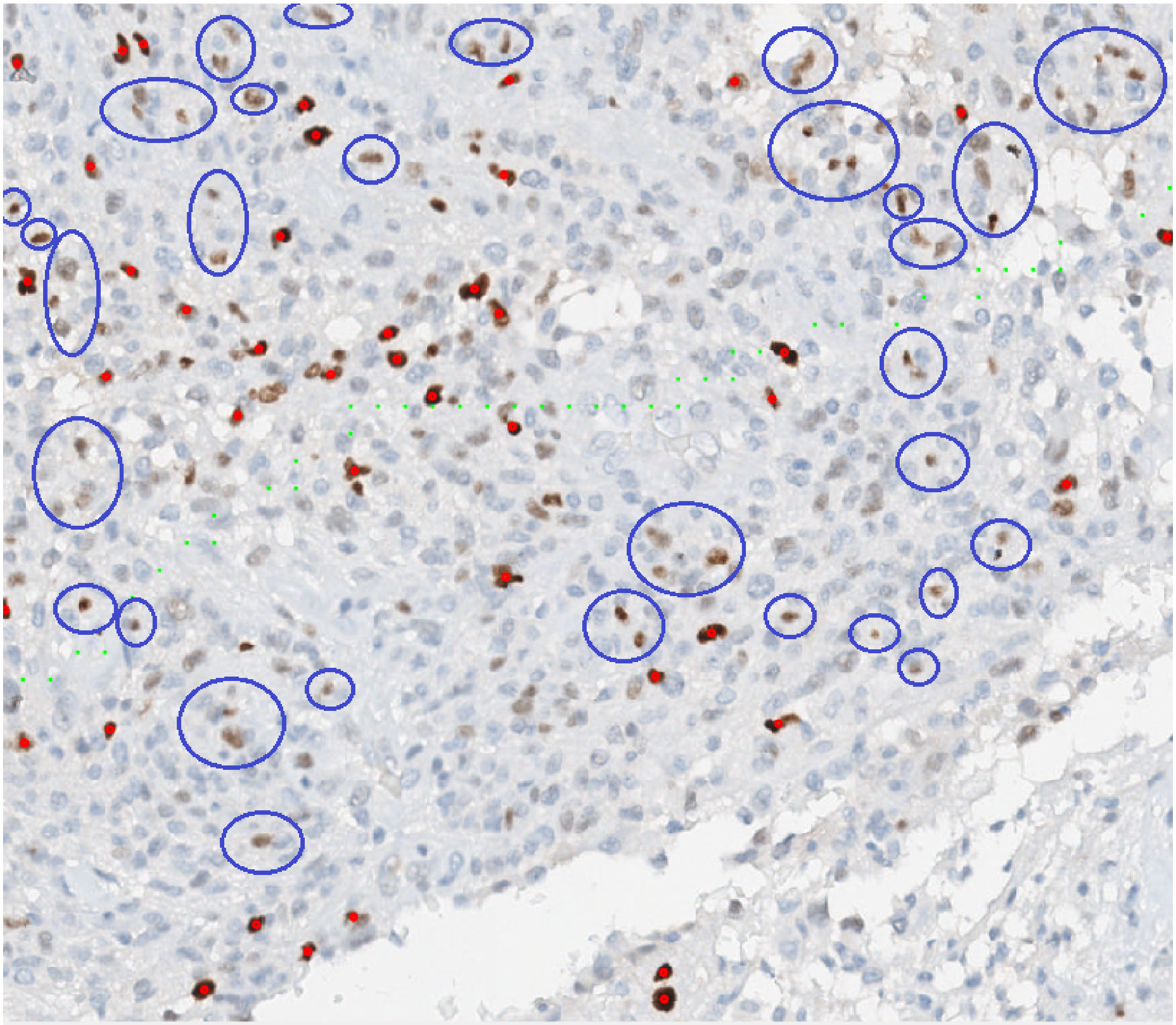

Figure 3. A small section of a large tumor sample. Cells beyond the threshold are shown with a small red circle. Ambiguous cells are circled in blue for illustration. The algorithm used for image analysis only selected the most unequivocally expressing cells.

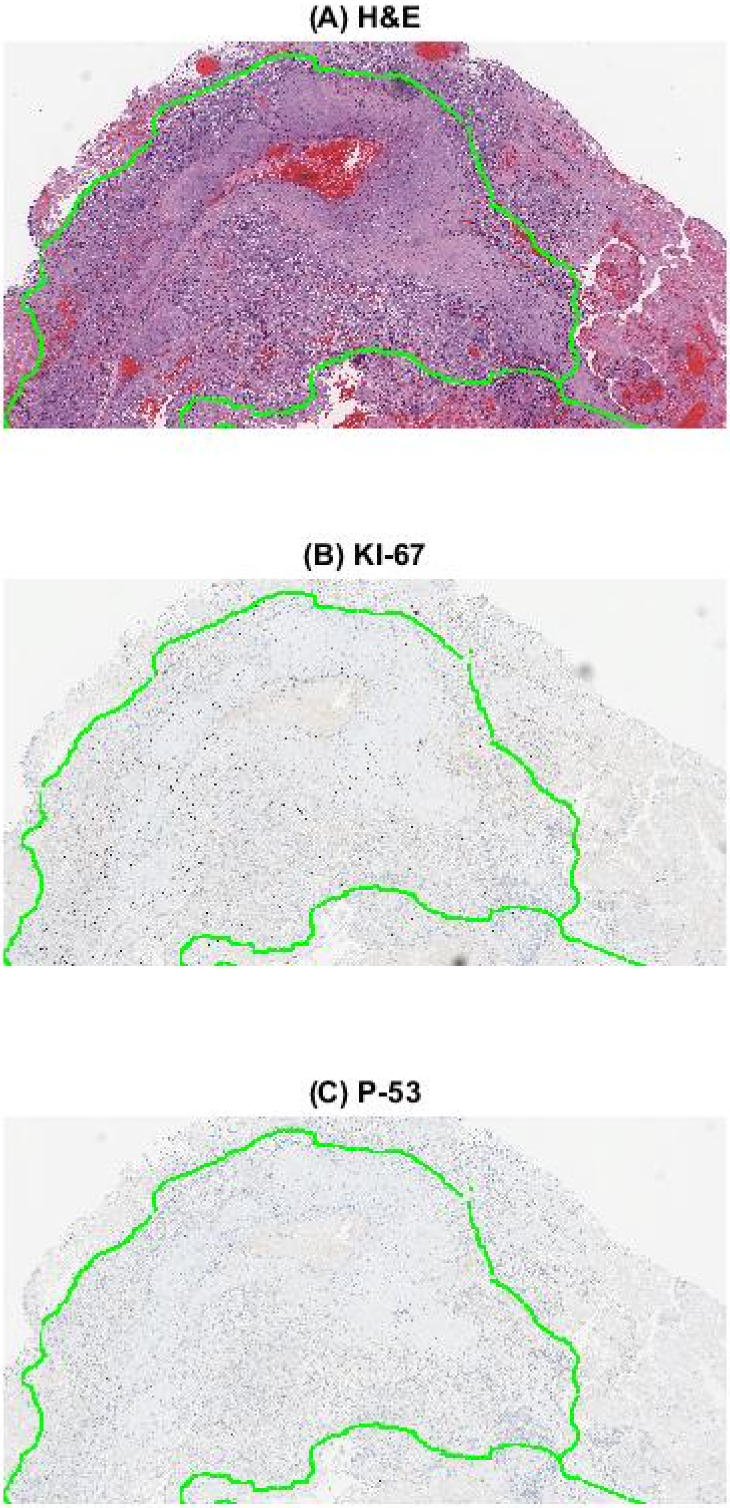

Figure 4. Breakdown of co-registered example shown in main text, figure 2. Green line in all depicts pathologist identified necrotic border.

Decoupled images from main paper figure 2

Depicted in figure 6.

References

1. Grimes, D. R. *et al.* Estimating oxygen distribution from vasculature in three-dimensional tumour tissue. *J. The Royal Soc. Interface* **13**, 20160070 (2016). DOI 10.1098/rsif.2016.0070.
2. Enderling, H. *et al.* Paradoxical dependencies of tumor dormancy and progression on basic cell kinetics. *Cancer research* **69**, 8814–8821 (2009).
3. Hockel, M. & Vaupel, P. Tumor hypoxia: definitions and current clinical, biologic, and molecular aspects. *J. Natl. Cancer Inst.* **93**, 266–276 (2001).
4. Grimes, D. R. *et al.* The role of oxygen in avascular tumor growth. *PLoS ONE* **11**, 1–19 (2016). DOI 10.1371/journal.pone.0153692.
5. Grimes, D. R., Kelly, C., Bloch, K. & Partridge, M. A method for estimating the oxygen consumption rate in multicellular tumour spheroids. *J. Royal Soc. Interface / Royal Soc.* **11**, 20131124 (2014). DOI 10.1098/rsif.2013.1124.
6. Brat, D. J. *et al.* Pseudopalisades in glioblastoma are hypoxic, express extracellular matrix proteases, and are formed by an actively migrating cell population. *Cancer research* **64**, 920–927 (2004).
7. Brat, D. J. & Mapstone, T. B. Malignant glioma physiology: cellular response to hypoxia and its role in tumor progression. *Annals internal medicine* **138**, 659–668 (2003).
8. Semenza, G. L. Hypoxia-inducible factor 1: oxygen homeostasis and disease pathophysiology. *Trends molecular medicine* **7**, 345–350 (2001).
9. Zagzag, D. *et al.* Expression of hypoxia-inducible factor 1α in brain tumors. *Cancer* **88**, 2606–2618 (2000).
10. Monteiro, A. R., Hill, R., Pilkington, G. J. & Madureira, P. A. The role of hypoxia in glioblastoma invasion. *Cells* **6**, 45 (2017).
11. Rong, Y., Durden, D. L., Van Meir, E. G. & Brat, D. J. 'pseudopalisading'necrosis in glioblastoma: a familiar morphologic feature that links vascular pathology, hypoxia, and angiogenesis. *J. Neuropathol. & Exp. Neurol.* **65**, 529–539 (2006).
